# Chemiresistors Based on Hybrid Nanostructures Obtained from Graphene and Conducting Polymers with Potential Use in Breath Methane Detection Associated with Irritable Bowel Syndrome

**DOI:** 10.3390/ijms25105552

**Published:** 2024-05-20

**Authors:** Alexandru F. Trandabat, Romeo C. Ciobanu, Oliver Daniel Schreiner, Thomas Gabriel Schreiner, Sebastian Aradoaei

**Affiliations:** 1 Department of Electrical Measurements and Materials, Gheorghe Asachi Technical University, 700050 Iasi, Romania; ftranda@tuiasi.ro (A.F.T.); oliver090598@yahoo.com (O.D.S.); schreiner.thomasgabriel@yahoo.com (T.G.S.); arsete@tuiasi.ro (S.A.); 2 Department of Medical Specialties III, Faculty of Medicine, University of Medicine and Pharmacy “Grigore T. Popa”, 700115 Iasi, Romania

**Keywords:** graphene, conducting polymers, chemiresistor, breath methane detection, irritable bowel syndrome

## Abstract

This paper describes the process of producing chemiresistors based on hybrid nanostructures obtained from graphene and conducting polymers. The technology of graphene presumed the following: dispersion and support stabilization based on the chemical vapor deposition technique; transfer of the graphene to the substrate by spin-coating of polymethyl methacrylate; and thermal treatment and electrochemical delamination. For the process at T = 950 °C, a better settlement of the grains was noticed, with the formation of layers predominantly characterized by peaks and not by depressions. The technology for obtaining hybrid nanostructures from graphene and conducting polymers was drop-casting, with solutions of Poly(3-hexylthiophene (P3HT) and Poly[(9,9-dioctylfluorenyl-2,7-diyl)-co-bithiophene] (F8T2). In the case of F8T2, compared to P3HT, a 10 times larger dimension of grain size and about 7 times larger distances between the peak clusters were noticed. To generate chemiresistors from graphene–polymer structures, an ink-jet printer was used, and the metallization was made with commercial copper ink for printed electronics, leading to a structure of a resistor with an active surface of about 1 cm^2^. Experimental calibration curves were plotted for both sensing structures, for a domain of CH_4_ of up to 1000 ppm concentration in air. A linearity of the curve for the low concentration of CH_4_ was noticed for the graphene structure with F8T2, presenting a sensitivity of about 6 times higher compared with the graphene structure with P3HT, which makes the sensing structure of graphene with F8T2 more feasible and reliable for the medical application of irritable bowel syndrome evaluation.

## 1. Introduction

Graphene, the thinnest and most resistant material, has extraordinary thermal conductivity and electronic mobility and has been the center of attention in recent years worldwide due to its exceptional characteristics with applicability in many fields. Graphene has been studied since the 1960s as monolayer graphite-on-metal substrates and even earlier as individual layers in graphite intercalation compounds. The first electrical measurements on monolayer graphene were published in 2004 [1], sparking interest in the fabrication of isolated samples by mechanical exfoliation of graphite. In order to make large volumes of devices, it is necessary to obtain graphene on large surfaces that are easy to handle and with as few defects as possible; an essential condition for the performance of the devices. There are different techniques for producing monolayer graphene but the most popular method at the moment is the so-called chemical vapor deposition (CVD) [2] on a Ni or Cu film as a catalyst [3,4,5,6,7,8]. Using this method, large graphene surfaces—mono-bi- or multi-layered—of relatively high quality can be produced. The benefits of using CVD for the deposition of materials on the substrate include the very good quality of the resulting material, represented by impermeability, high purity, fine grain, and increased hardness compared to other coating methods. A major problem that the scientific world is still trying to solve is that although it is possible to obtain high-quality graphene on a substrate using CVD, successfully separating or exfoliating the graphene from the substrate proves to be more complicated because the bond between the graphene and the substrate is not yet fully understood. It is not easy to achieve the separation without damaging the graphene structure or the properties of the material. Separation techniques differ depending on the type of substrate used. Traditional delamination methods for graphene transfer use corrosive substances to remove the substrate which, in addition to high costs, produces polluting residues for the environment and dissolution of the substrate. Such techniques limit applicability at the industrial level. A feasible method is to obtain graphene by CVD on a Cu substrate. During the reaction that takes place between the Cu substrate and graphene, a high hydrostatic compression is created, coupling the graphene to the substrate [9]. It has been found possible, however, to intercalate a layer of copper oxide (which is mechanically and chemically weak) between the graphene and the substrate to reduce this pressure and allow the graphene to be removed relatively easily at low cost and without harmful chemicals, and the substrate can be even reused [10]. Electrochemical delamination can also use a non-polluting electrolyte resulting in the separation of, e.g., polymethyl methacrylate (PMMA)/graphene film from the substrate, which can be reused to obtain graphene. In this way, a non-destructive transfer of graphene from the metal substrate can be achieved [11,12]. In [13], a “bubble-free” transfer method was developed to avoid mechanical damage, i.e., the removal of the oxide layer formed by the infiltrated air at the graphene/Cu interface, resulting in a lower percentage of defects.

By its specific properties, graphene is targeting the global challenges in transparent electrodes, field-effect transistors, flexible touch screens, sensors for single-molecule gas detection, superconductivity, DNA sequencing, etc., as described in [14,15,16] for example. In the last few years, hybrid structures made by graphene with different polymers have largely been studied. In [17], a technology for obtaining graphene/polyaniline, graphene/poly(3,4 ethyldioxythiophene), and graphene/polypyrrole(PPy) nanocomposites is emphasized. In [18], a vast description of different hybrid structures including metallic oxides, graphene, and conducting polymers such as polyindole, polypyrrole, and polyaniline, is presented. In [19], an introduction to similar structures of graphene oxide/conducting polymer composites, this time as hydrogels, is offered. Other descriptions of similar technologies may be found in [20,21,22,23,24,25]. The development of such hybrid nanostructures is related to their special semiconducting features, exploitable for micro-electronic and/or electrochemical applications. 

The first main application of the hybrid structures obtained from graphene and conducting polymers is related to sensors. In the last 15 years, various types of sensors have been developed, starting from the simplest ones, e.g., for humidity [26,27], temperature [28], gas detection [29,30,31,32,33], including waste gas evaluation [34] or other types of chemical sensors [35,36]; continuing with biosensors with different applications for the detection of dopamine, serotonin, cholesterol, bilirubin, uric acid, etc. [37,38,39,40,41,42]; dedicated sensors for environmental monitoring by the detection of pollutants in water, including heavy ions [43,44]; finalizing with food and drug analyses [45,46]. The second main application of the hybrid structures obtained from graphene and conducting polymers, occurring mainly in the last 10 years, is related to photovoltaic energy generation and energy storage applications/supercapacitors [47,48,49] as well as other photocatalytic applications [50].

In line with the above-described applications of bio-sensors, our paper intends to investigate the base of a new type of chemiresistors, with potential use in breath methane detection associated with irritable bowel syndrome. Hydrogen/Methane breath testing [51,52] is a widely used diagnostic tool based on the concept that some specific gases represent by-products of faulty fermentation, beyond the ones assured by gut microorganisms. On the other hand, the prevalence of irritable bowel syndrome, characterized by inflammation of the gastrointestinal tract—which has become a common disorder nowadays due to exposure to pollutants, food additives, and stress—represents a day-by-day preoccupation of many subjects, affecting their quality of life. Glucose, lactose, and fructose are normally absorbed mainly in the small intestine, and further in the colon; increased gas production following their ingestion is associated with malabsorption or premature fermentation due to excessive bacteria activity, micro-gas formation, and chemical attack at the level of intestinal cells. Consequently, methane gas is absorbed from the gastrointestinal tract, exhaled via the lungs, and is potentially measurable in breath. Increased gas production may predict small intestinal bacterial overgrowth, a precursor to irritable bowel syndrome. Secondly, such a phenomenon may be also related to intolerances to some food or food allergies, aspects which largely extend the importance of the use of such sensors. Unfortunately, detection of methane in the breath is challenging due to relatively small concentrations and inherent interferents, which is why only a few studies have been conducted in this direction but no commercial sensor has been developed to date.

Resistive gas sensors are versatile and cost-effective solutions for detecting a wide range of gases in diverse applications. Such sensors have a much simpler design, which allows for mass production and facile integration within signal processing systems. Using an adequate choice of sensing material, resistive gas sensors can be tailored to detect a specifically targeted gas, in our case methane [53,54,55]. On the other hand, they have reduced selectivity and longer response and recovery times [55,56,57]. Factors such as temperature and humidity may impact the performance of resistive gas sensors [58]. 

The importance of this research consists in the development of a simple and feasible concept of a resistive gas sensor based on graphene—conducting polymer assemblies, for the detection and evaluation of methane in breath—which can be related to the real occurrence and severity of irritable bowel syndrome. The sensor principle presented in the paper is much simpler, cost-effective, and more efficient compared to the homolog methods used nowadays [59]. It is considered that for the purpose of preliminary investigations related to irritable bowel syndrome, or for periodic checks at home, under room temperature conditions, the proposed chemiresistor can respond in a feasible way as long as the measurements are not taken in quick succession, and the syndrome detection is based on exceeding a pre-defined threshold value and not requiring a very exact assessment of the exhaled gas concentration.

## 2. Technology for Obtaining Graphene on Copper Substrate

### 2.1. Materials and Preparation Methods

The technology of graphene dispersion and support stabilization on chemical vapor deposition (CVD) equipment was based on the use of the AS-One 100 HT Rapid Thermal Processor installation (ANNEALSYS, Montpellier, France) placed in a clean room laboratory ISO 7 [60]. To obtain graphene, Cu foils (purity 99.9%) with dimensions of 2 × 2 cm and a thickness of 25 µm were used as substrates. Initially, the Cu foils were subjected to successive steps of ultrasonic cleaning in acetone and isopropyl alcohol (immersion time in each solvent being 10 min). The ultrasound was performed in an Elmasonic S 10 H ultrasound bath. After the cleaning step, the Cu foils were introduced into the CVD installation in order to deposit the graphene layers. The use of graphite as a susceptor has several advantages including good mechanical properties, thermal conductivity at high temperatures, and a low level of metal impurities. To further increase the purity, the susceptor was coated with a layer of silicon carbide (SiC) through a CVD process. The maximum temperature at which the SiC-coated graphite susceptor can be used is up to 1250 °C. Because SiC-coated graphite susceptors are sensitive to temperature gradients, low heating rates were used, especially for temperatures lower than 700 °C. After the cleaning process, the Cu foils were introduced into the working area of the CVD installation on the surface of the SiC-coated graphite susceptor. The process started with successive steps for cleaning the work area (pumping and purging) using Argon. Next, the preliminary pump was started up to 10 mBar in an atmosphere of Hydrogen. The process temperatures were 900 °C and 950 °C, respectively. To reach these temperatures, several heating and stabilization steps were used (250, 300, 500, and finally 900 °C) at a heating rate of 5 °C/s to extend the life of the susceptor. Finally, to reach the temperature of 900 °C, for good stabilization of the process, a 900 s duration was needed. Next, after the temperature was stabilized at 900 °C, the Cu substrate underwent treatment for 1800 s in an atmosphere of hydrogen during which the graphene layers formed on the crystallites of the Cu. After the process was finished, a 10-min pause was included before opening the work area for the susceptor to cool down. After cooling, the sample of Cu substrate covered with a graphene layer was taken with the help of tweezers, positioned on a special support in the clean room in the chemical niche, and submitted to a cleaning process with acetone and isopropyl alcohol.

To transfer the graphene to the substrate of interest, the following steps were carried out: First, a PMMA layer of approximately 600 nm was deposited by spin-coating at a speed of 3000 rpm for 60 s; second, a thermal treatment of the PMMA/graphene/Cu assembly at 100 °C for 20 min was applied, on a hot plate, resulting in the strengthening of the PMMA.

The next technological step was represented by the electrochemical delamination by use of a PARSTAT 4000 potentiostat (AMETEK Scientific Instruments Inc., Oak Ridge, TN, USA) with the related software. A cell with three electrodes and 0.5 M NaCl solution was used, i.e., a working electrode—the PMMA/graphene/Cu assembly—a calomel reference electrode (SCE), and a counter electrode—a Pt plate. A potential of −l.4 V was applied at the SCE, and after about 5 min, the detachment of graphene from the edges of the Cu substrate was observed. After about 7 min, the graphene was completely detached and the PMMA/graphene assembly floated. The assembly was further extracted by immersing it in a solution at 45 °C and transporting it in a vessel with demineralized water. After collecting the assembly, and drying (with a very weak nitrogen jet and then in dry air), acetone was used to dissolve the PMMA layer—a process that normally takes about 1 h—and finally, it was immersed in isopropyl alcohol to clean the sample of any debris and dried with a nitrogen jet. The samples were finally transferred onto a SiO_2_/Si substrate.

### 2.2. Characterization Equipment

Scanning electron microscopy (SEM) was performed with Lyra III XMU equipment (TESCAN GROUP a.s., Brno-Kohoutovice, Czech Republic). A progressive morphological analysis was performed to evaluate the obtained graphene layer.

Atomic force microscopy (AFM) optical analysis was performed with a Dimension Edge unit (Bruker, Billerica, MA, USA). The roughness evaluation was conducted with the following derived parameters: Ra = Roughness Average; R_Sk_ = Skewness; RMS = Root Mean Square Roughness; R_Ku_ = Kurtosis. The results for the roughness parameters are presented as average for 4 scanned zones on each sample type.

### 2.3. Results and Discussion

#### 2.3.1. SEM Analysis

**Figure 1:**
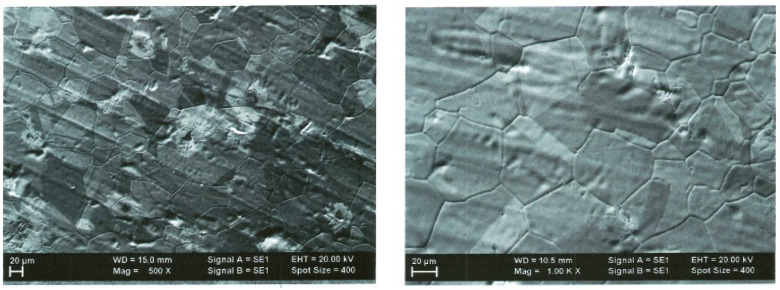
SEM images at 500× and 1000× magnification process at T = 900 °C.

**Figure 2:**
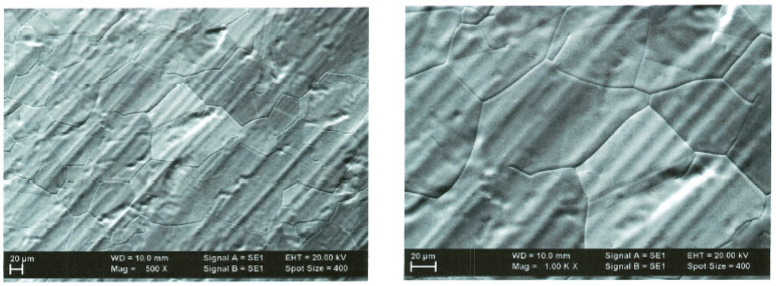
SEM images 500× and 1000× magnification process at T = 950 °C.

Figure 1 and Figure 2 show samples of graphene on Cu foil obtained by CVD at two temperatures, 900 °C and 950 °C, respectively. The difference in contrast is due to the number of monolayers in the obtained material.

In general, for both cases, the same morphology of grains—even when of different sizes—is present, with uniform distribution over the surface.

#### 2.3.2. AFM Analysis

**Figure 3:**
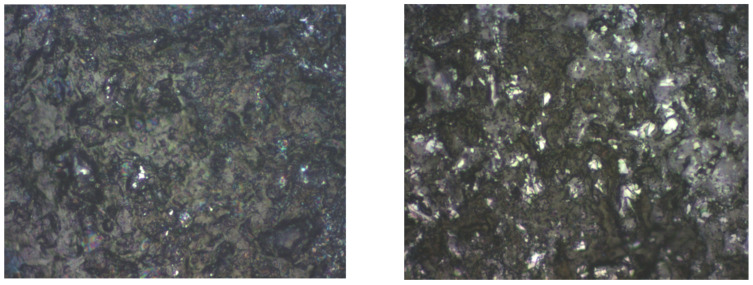
Optical analysis at 100× magnification process at T = 900 °C and T = 950 °C, respectively.

The AFM optical analysis shows the grain dimension, their distribution vs. surface, and the general roughness of the surfaces. The comparative optical analysis is presented in Figure 3. 

**Figure 4:**
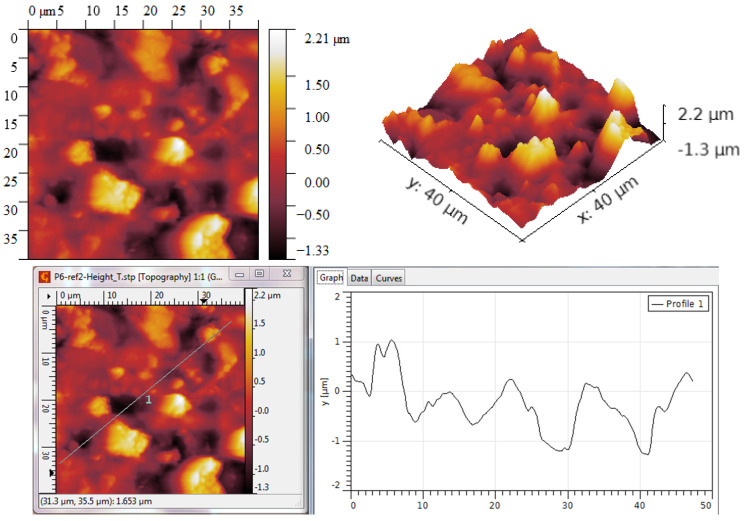
AFM Topographic 2D and 3D images and profile lines—process at T = 900 °C.

For the process at T = 900 °C, the grain size exceeds 2 μm, as seen in Figure 4. The grains are generally arranged either in smaller clusters or in slightly larger clusters, leading to the formation of zones characterized by slightly different R_ku_ or R_sk_ parameters but the coherence of statistical parameters led to the conclusion of a symmetric distribution of grains.

The R_ku_ value is above three, which means that the grains are placed such that they do not form depressions between them, and the R_sk_ value is also relatively high, which also suggests that no large depressions are formed, and hence more dense peaks are occurring, Table 1.

**Figure 5:**
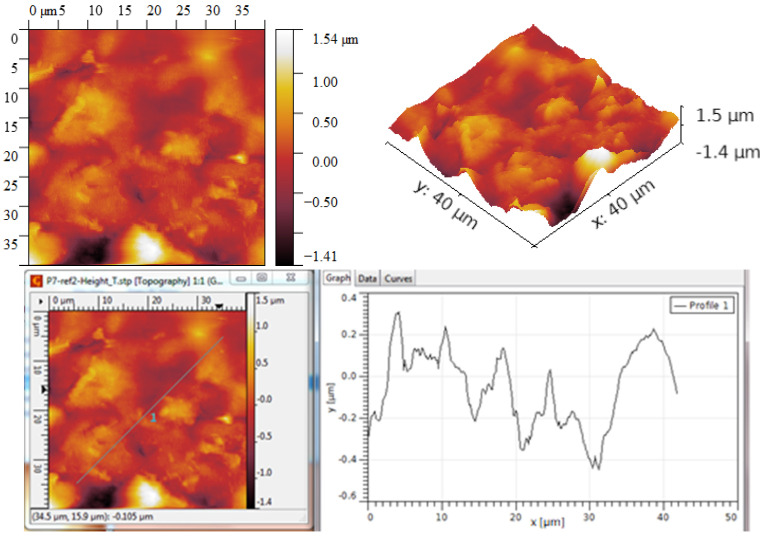
AFM Topographic 2D and 3D images and profile lines—process at T = 950 °C.

For the process at T = 950 °C, the grain size is lower even if they exceed 1.5 μm, as seen in Figure 5. The grains are generally arranged mainly in larger clusters, leading to the formation of zones characterized by slightly different R_ku_ or R_sk_ parameters. Also, in this case, the coherence of statistical parameters led to the conclusion of a symmetric distribution of grains.

The R_ku_ value is above three, which means that the grains are placed such that they do not form large depressions between them, Table 2. The R_sk_ value is lower compared to the process at T = 900 °C, which suggests that more depressions are formed but they are not so deep. The density of peaks is lower, leading to a better balance between peaks and depressions.

In all, the process at T = 950 °C led to graphene structures characterized by RMS and R_a_ roughness parameters with lower values compared to the graphene structures obtained at T = 900 °C. This decrease may be due to a better settlement of the grains. Beyond this, the values of the parameters R_ku_ and R_sk_ indicate, in both cases, the formation of layers predominantly characterized by peaks and not by depressions, which means that the grains settle in such a way that they do not form holes between them. Accordingly, the graphene structures are uniform and without structural defects.

## 3. Technology for Obtaining Hybrid Nanostructures from Graphene and Conducting Polymers

### 3.1. Materials and Preparation Methods

Graphene structures obtained at T = 950 °C were chosen due to their lower values of roughness and better settlement of the grains compared to the graphene structures obtained at T = 900 °C. The technology for obtaining hybrid nanostructures from graphene and conducting polymers was drop-casting, and five samples of each type were manufactured for comparison of technological feasibility.

In the case of Poly 3-hexylthiophene (P3HT), 15 mg/mL of polymer was dissolved in CHCl_3_ at room temperature in an ultrasonic bath and kept for 30 min for uniform dispersion.

In the case of Poly[(9,9-dioctylfluorenyl-2,7-diyl)-co-bithiophene] (F8T2), 20 mg/mL of polymer was dissolved in toluene at 60 °C in an ultrasonic bath, and kept for 30 min for uniform dispersion.

In both cases, 120 μL of each polymer solution was deposited on graphene (SiO_2_/Si substrate) by the drop-casting method using Pasteur pipettes. The evaporation of each solvent took place for 30 min in vacuum, using a Pfeiffer vacuum pump connected to a desiccator.

### 3.2. Results and Discussion

#### 3.2.1. Hybrid Nanostructures from Graphene and P3HT

**Figure 6:**
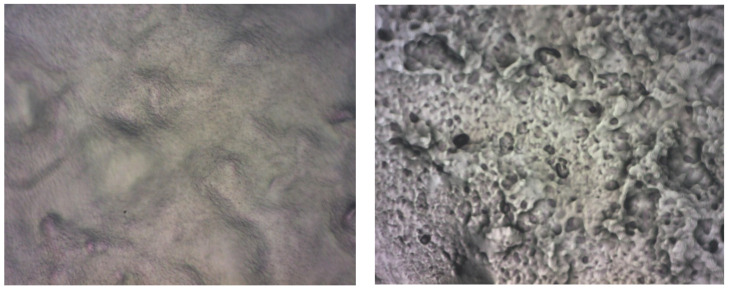
Optical analysis of graphene–P3HT at 100× and 500×.

For graphene covered with P3HT, a different topography is observed by AFM analysis compared to the graphene structures. Although, at first glance, at 100× it seems to have a fairly uniform grain distribution, at 500× it can be noticed that the roughness is quite high and the grains are arranged in different modes, Figure 6. Grains of different sizes but also smoother stretches can be observed.

**Figure 7:**
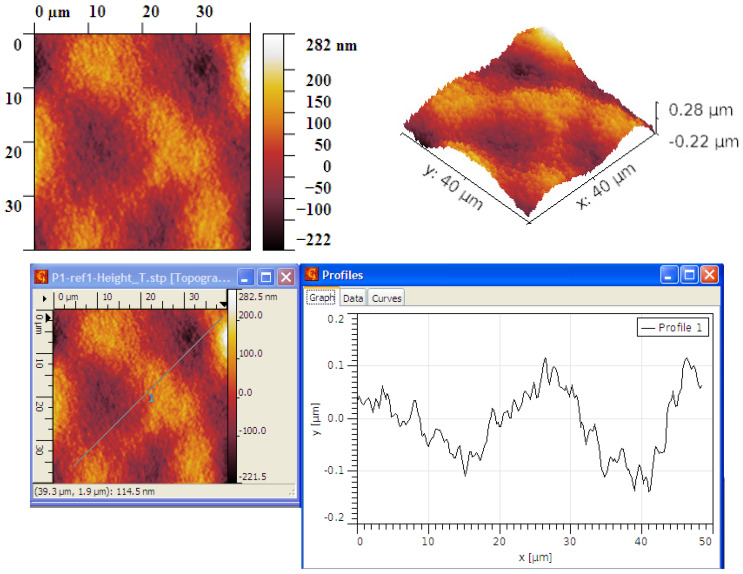
AFM Topographic 2D and 3D images and profile lines for graphene–P3HT.

The grain size is low, generally under 0.3 μm, as seen in Figure 7. The grains are generally arranged in larger clusters. The R_ku_ values are not very high, still around three, which means that the grain distribution is quite symmetrical. The R_sk_ values are low, even lower compared to graphene, indicating that more depressions are formed by polymer deposition, even if not so deep (no pits have been formed). In this case, we can estimate an about equal percent of peaks and depressions spread upon the surface, Table 3.

#### 3.2.2. Hybrid Nanostructures from Graphene and F8T2

**Figure 8:**
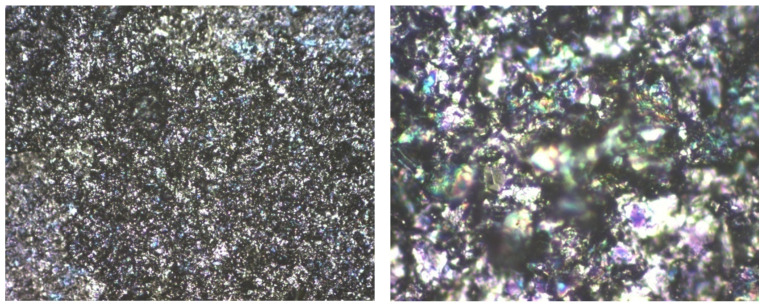
Optical analysis of graphene–F8T2 at 100× and 500×.

For graphene covered with F8T2, a different topography is observed compared to graphene structures too. Analyzing Figure 8, at 500×, it seems to have a fairly uniform grain distribution but the roughness is high, much higher compared to the graphene deposited with P3HT. The grains are arranged less uniformly, and there are grains of different sizes separated by smoother stretches.

**Figure 9:**
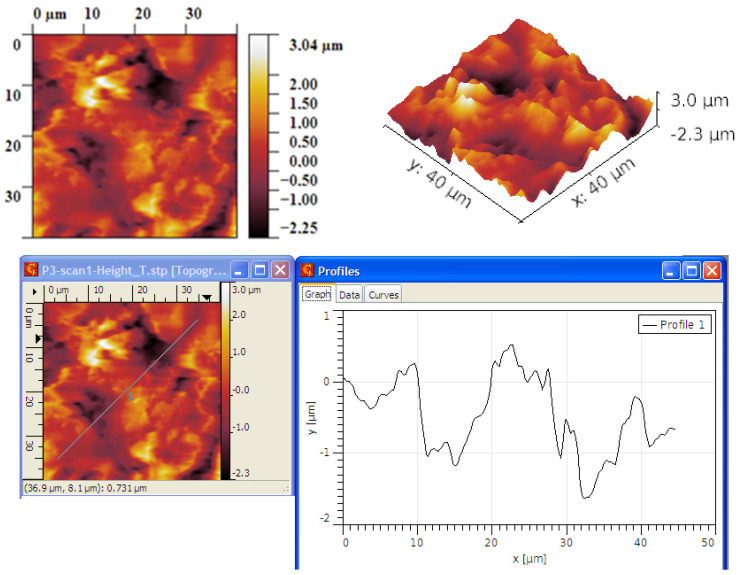
AFM Topographic 2D and 3D images and profile lines for graphene–F8T2.

The grain size is generally 3 μm, about 10 times larger compared to the graphene deposited with P3HT, as seen in Figure 9. The grains are generally arranged in smaller clusters. The R_Sk_ values are low, indicating that some depressions are formed by polymer deposition but the depressions architecture is dispersed, and in general the grains settle without leaving too much free space between them. The R_ku_ values are not very high, still around three, which means that the grain distribution is quite symmetrical, Table 4.

In all, AFM emphasized a higher roughness in the case of F8T2 compared to P3HT and a much larger dimension of grain size. In both cases, a quite symmetrical distribution of grains was noticed, with reduced free space between them. Such structures with symmetrical distribution and roughness dimension at a micrometer-scale are considered optimal for the application as gas sensors.

## 4. Analysis of Functionality as Gas Sensors for Methane

In the literature, different processes of metallization of graphene-supported composite materials are described, e.g., in [61], most of them are inadequate for simple sensor purposes. In our case, an ink-jet printer was used, and the metallization was made with commercial copper ink for printed electronics. A structure of a resistor was generated, with an active surface of about 1 cm^2^, limited by two metalized areas forming the conductive connections. Its functionality as a gas sensor was tested using an experimental system, similar to the one described in [62]. The sensor was introduced in a closed enclosure, which only allowed the exchange of gases by two valves and access to the electrical connections. The resistance of the sensor was measured externally, by a precision ohmmeter. Variable mixtures of CH_4_ in synthetic air (80% nitrogen and 20% oxygen) were passed through the closed enclosure through one of the valves and let free on the other, to maintain a pressure of 1 atm. The exact content of CH_4_ in synthetic air was separately analyzed, sample by sample, by use of a 7890 portable combustible gas detector (Seitron SpA, Mussolente, Italy) in order to correlate the resistance and CH_4_ concentrations on the calibration curves.

**Figure 10:**
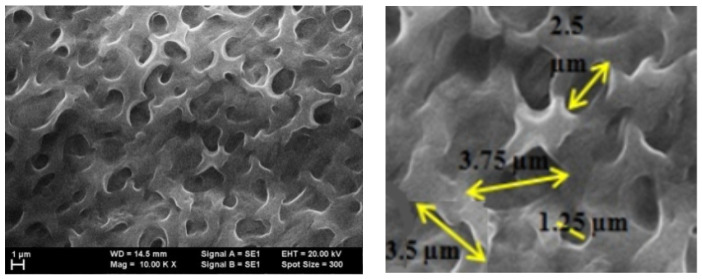
SEM image for graphene–P3HT structure.

**Figure 11:**
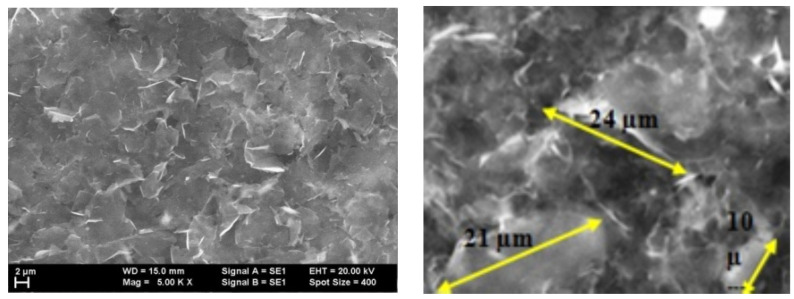
SEM image for graphene–F8T2 structure.

Due to the large difference in roughness (grain size), the behavior of both graphene structures with F8T2 and with P3HT deposition were comparatively analyzed for their potential features on methane detection. As observed in Figure 10 and Figure 11, the distribution of the cavities between peaks is different but still uniformly dispersed. In these figures, some distances between the peak clusters are marked with yellow arrows. In the case of the graphene–F8T2 structure, the distances between the peak clusters are about 7 times larger compared to the graphene–P3HT structure (e.g., about 20 μm compared to about 3 μm), under the circumstances that also the peaks were found about 10 times higher (Figure 7 and Figure 9). Consequently, any potential difference in sensitivity of the developed sensing structures can be explained further by this spatial architecture.

**Figure 12:**
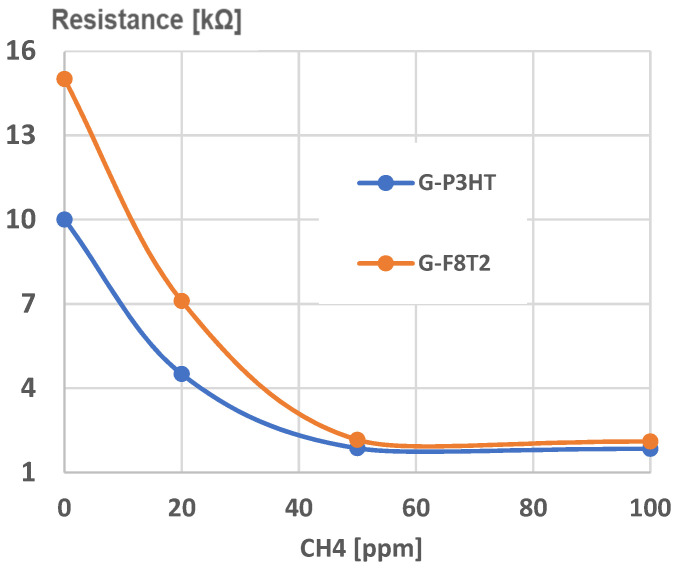
Limit of detection for sensing CH_4_.

Experimental calibration curves were plotted for both sensing structures (Graphene-P3HT/G-P3HT and Graphene-F8T2/G-F8T2) for a larger domain of CH_4_, concentration in air, of up to 1000 ppm CH_4_. The limit of detection (LoD) was found as 50 ppm, a very reasonable value for many potential applications, as presented in Figure 12.

Under this value of CH_4_ concentration in air, the resistance of both sensors presents extremely high values, with low credibility to be put in correlation with lower values of gas concentration.

**Figure 13:**
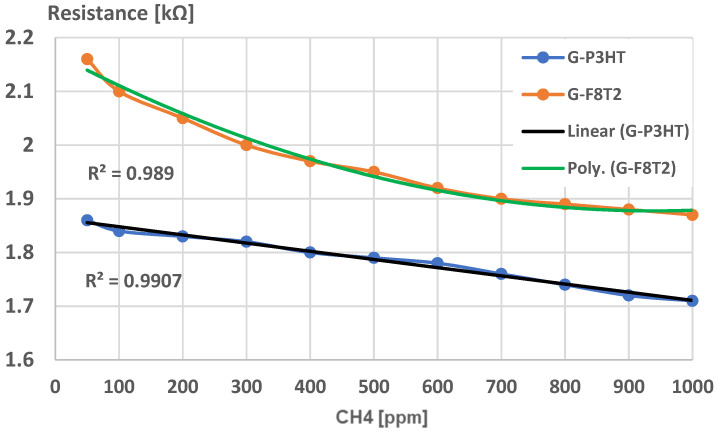
Experimental calibration curve for sensing CH_4_ for general use.

A preliminary experimental calibration curve for sensing CH_4_ for general use is presented in Figure 13. A high degree of correlation can be noticed in both cases. The curve for the graphene structure with P3HT presents a high linearity and lower values of resistance, which makes it useful for large-scale determination of CH_4_ concentrations in air when using a simple signal processing system. By comparison, the curve for the graphene structure with F8T2 may be approximated with a polynomial curve of at least second degree, which makes the signal processing approach more difficult, and, consequently, may increase the sensor cost. On the other hand, the slope of the characteristic for the graphene structure with P3HT is low, an aspect that indicates a lower sensitivity of the sensor. The sensor sensitivity can be clearly put in relation to the active surface of the sensor exposed to the targeted gas, and in our case, the surface architecture of the graphene structure with F8T2 presents a larger active surface due mainly to higher and more dense peaks, as noticed in Figure 9 and Figure 11.

**Figure 14:**
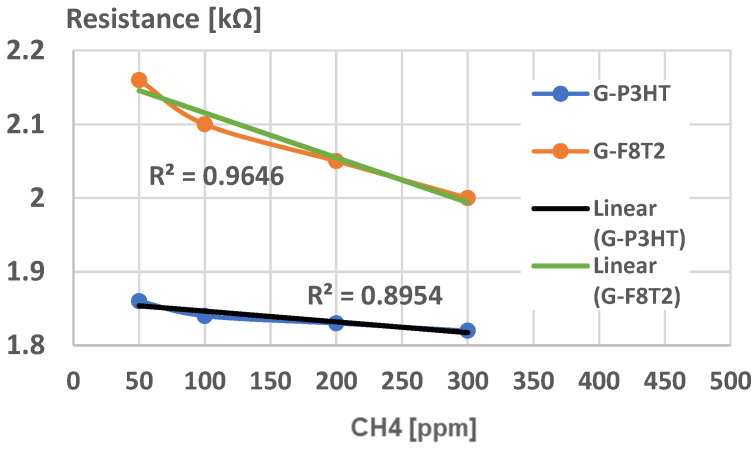
Experimental calibration curve for sensing CH_4_ for medical use.

But, if the application for testing the breath methane detection associated with irritable bowel syndrome is targeted, lower CH_4_ concentrations in air must be detected, with a threshold value of, e.g., 100 ppm, which may indicate the syndrome occurrence [62,63]. In this case, a new experimental calibration curve was analyzed, Figure 14. Here, one can notice that both curves have a high degree of linearity for this CH_4_ concentration domain but the slope for graphene structure with F8T2 is 6 times higher compared with the graphene structure with P3HT, which makes this structure more sensitive, feasible, and reliable for medical application. The inferior limit of CH_4_ concentration detection, here 50 ppm, is considered enough when taking into account the correlation of CH_4_ production with the severity of irritable bowel syndrome because lower concentrations do not particularly indicate a real occurrence of irritable bowel syndrome, [62,63]. In some studies, e.g., as described in [64,65], lower concentrations of CH_4_ (20–40 ppm) were also analyzed but only for the purpose of detecting specific intestinal bacterial overgrowth, which may eventually influence the occurrence of irritable bowel syndrome; however, this approach was not the purpose of this paper as it targets the already established irritable bowel syndrome.

**Figure 15:**
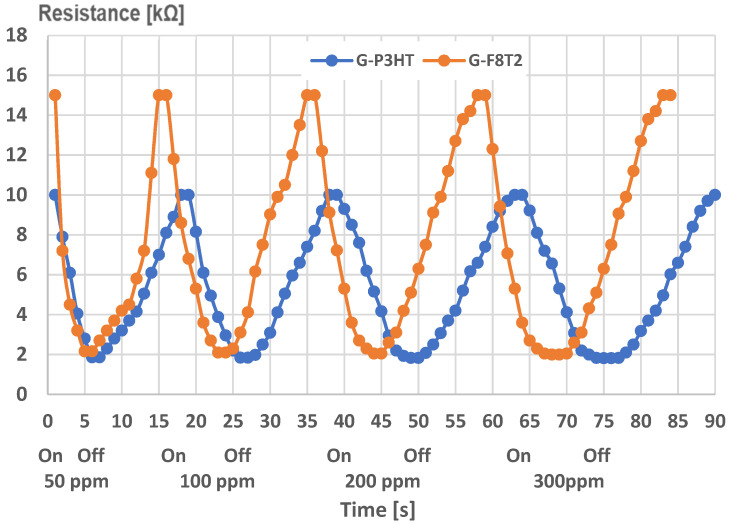
Experimental resistance–time curves for the sensing structures.

A final comparative analysis of the sensing structures of graphene with P3HT, and, respectively, with F8T2, is presented in Figure 15, indicating the resistance–time evolution when measuring four different concentrations of CH_4_ (50, 100, 200, and 300 ppm). “On” marks the moment when starting the measurements with CH_4_ and synthetic air-tailored mixtures, till the stationary value of resistance is obtained, as indicated in Figure 14. “Off” indicates the moment when only synthetic air is sent to the sensor, till it reaches the initial value of resistance in air. Both resistance decrease and restoration display a quasi-exponential characteristic. At first view, the increased sensitivity of the structure of graphene with F8T2 is noticed, leading to a quicker response.

**Figure 16:**
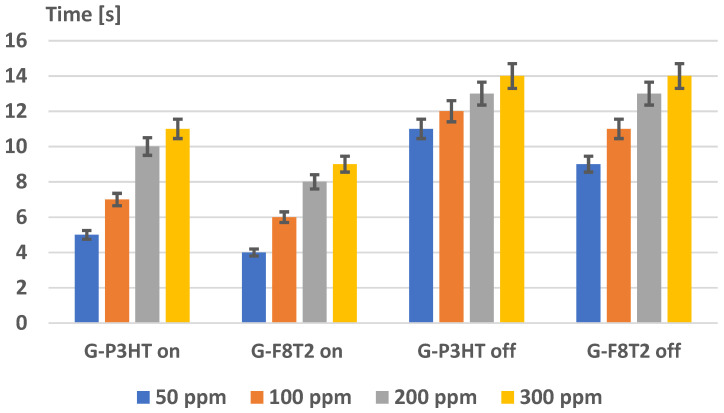
Experimental response (on) and recovery time (off) for the sensing structures.

The evaluation of the experimental response (on) and recovery time (off) for the sensing structures is presented in Figure 16. It was noticed that, in general, both response and recovery time values are lower for the structure of graphene with F8T2. The difference is even much higher at lower concentrations of CH_4_ (50, 100 ppm). An interesting phenomenon occurs at higher concentrations of CH_4_ (200, 300 ppm) regarding the recovery time when both structures seem to reach the same values, exceeding 12 s.

The obtained values of response time of about 4 s for the structure of graphene with F8T2, at a concentration of CH_4_ of 50 ppm, is very reliable in the quick detection of irritable bowel syndrome, being associated with a relatively quick exhalation of air through the mouth. Once the syndrome is detected, its severity can be further reevaluated by a slow exhalation of air, of about 9 s, which is reasonable as a procedure. As regards the recovery time value, it is considered also feasible because even at a higher concentration of CH_4_ of, e.g., 300 ppm, it takes only about 14 s for the sensor to recover its initial resistance, and, for medical use, to wait about 1 min between two measurements is quite reasonable, even if needing to use the same device to evaluate more patients.

The response and recovery time values for the developed sensing structures are in line with other homolog gas sensors, e.g., based on semiconductive assemblies, as in [32,34,66,67] but in our case, the response time values are lower due to the direct use and higher conductivity of graphene-conducting polymers assemblies. The sensor characteristic is superior to, e.g., [54], regarding the minimum detection limit, and can be tailored for different threshold values of CH_4_ concentrations in air, depending on the type of investigation and syndrome extent. In all, the use of a simple, low-value, and robust device for individual use is beneficial at the patient level because the syndrome evolution or treatment efficiency can be more effectively surveyed. Due to these successful results, even if preliminary, the sensor features will be further analyzed in the presence of perturbing factors, determined also by the breathing process, i.e., the potential influences of exhaled CO_2_ and exhaled humidity.

## 5. Conclusions

This paper describes the process of producing chemiresistors based on hybrid nanostructures obtained from graphene and conducting polymers.

The technology of graphene dispersion and support stabilization was based on the chemical vapor deposition technique. The transfer of the graphene to the substrate of interest was made by spin-coating of PMMA and further thermal treatment of the PMMA/graphene/Cu, followed by an electrochemical delamination. The samples were finally transferred onto a SiO_2_/Si substrate for microscopy analysis. The process at T = 950 °C led to graphene structures characterized by RMS and Ra roughness parameters with lower values compared to the graphene structures obtained at T = 900 °C. A better settlement of the grains was noticed, with the formation of layers predominantly characterized by peaks and not by depressions.

The technology for obtaining hybrid nanostructures from graphene and conducting polymers was drop-casting, with solutions of P3HT and F8T2. AFM analysis emphasized a higher roughness in the case of F8T2 compared to P3HT, with about a 10 times larger dimension of grain size. In both cases, a quite symmetrical distribution of grains was noticed, with reduced free space between them. SEM analysis emphasized that the distribution of the cavities between peaks are different but still uniformly dispersed for both polymers; however, in the case of the graphene–F8T2 structure the distances between the peaks clusters are about 7 times larger compared to graphene—P3HT structure.

To generate chemiresistors from graphene–polymer structures, an ink-jet printer was used, and the metallization was made with commercial copper ink for printed electronics. A structure of a resistor was generated, with an active surface of about 1 cm^2^. Experimental calibration curves were plotted for both sensing structures, for a larger domain of CH_4_ concentration in air, of up to 1000 ppm CH_4_. The limit of detection was found to be 50 ppm. The curve for the graphene structure with P3HT presents a high linearity and lower values of resistance, which makes it useful for large-scale determination of CH_4_ concentrations in air, by use of a simple signal processing system.

For testing the breath methane associated with irritable bowel syndrome, only lower CH_4_ concentrations in air must be detected, with a threshold value of, e.g., 100 ppm, which may indicate the syndrome occurrence. The linearity for this CH_4_ low concentration domain was noticed also for the graphene structure with F8T2, and, more than this, the respective slope was found to be 6 times higher compared with graphene structure with P3HT, which makes the sensing structure of graphene with F8T2 more feasible and reliable for the medical application for irritable bowel syndrome assessment.

## Figures and Tables

**Table 1:** Average roughness parameters determined by AFM lines—process at T = 900 °C.

Scanned Area	RMS (nm)	Ra (nm)	R_Sk_	R_Ku_
40 × 40 μm	489	376	0.447	3.63

**Table 2:** Average roughness parameters determined by AFM lines—process at T = 950 °C.

Scanned Area	RMS (nm)	Ra (nm)	R_Sk_	R_Ku_
40 × 40 μm	339	243	0.261	4.26

**Table 3:** Average roughness parameters determined by AFM lines—graphene, P3HT.

Scanned Area	RMS (nm)	Ra (nm)	R_Sk_	R_Ku_
40 × 40 μm	64	53	0.143	3.31

**Table 4:** Average roughness parameters determined by AFM lines—graphene–F8T2.

Scanned Area	RMS (nm)	Ra (nm)	R_Sk_	R_Ku_
40 × 40 μm	705	546	0.191	3.36

## Data Availability

Data are contained within the article.
